# Baltimore College of Dental Surgery

**Published:** 1867-03

**Authors:** 


### BALTIMORE COLLEGE OF DENTAL SURGERY.

The Twenty-seventh Annual Commencement of this institution was held on the evening of Feb. 28th. The usual exercises, prayer, addresses, etc., were appropriately conducted, and the degree of "Doctor of Dental Surgery" was conferred on thirty-one candidates, of whom Maryland furnished 10, Virginia 6, Alabama 3, Pennsylvania and Indiana, each 2, South Carolina, Florida, North Carolina, Tennessee, Louisiana, Mississippi, District of Columbia, and France, each 1. We are glad to see such evidence of prosperity on the part of this institution. W.

